# HEARING LOSS AND HEALTH CARE SATISFACTION

**DOI:** 10.1093/geroni/igz038.167

**Published:** 2019-11-08

**Authors:** Nicholas Reed, Nicholas Reed, Amber Willink, Jennifer Deal, Frank R lin

**Affiliations:** Johns Hopkins University, Baltimore, Maryland, United States

## Abstract

Hearing loss (HL) impacts two-thirds of adults over 70 years and affects patient-provider communication which could limit satisfaction. We used two cross-sectional cohorts, The Atherosclerosis Risk in Communities Study (ARIC, n=250) and the Medicare Current Beneficiaries Survey (MCBS, n=12,311) to examine the relationship between HL (subjective and objective measures) and self-report satisfaction with quality of health care using multivariable-adjusted logistic regression. In ARIC, there was an interaction between HL and age such that HL had a greater impact on odds of dissatisfaction as age increased. In an 85-year-old, for every 10 dB increase in HL, the odds of being dissatisfied increased 1.33 (95% Confidence Interval [CI]:0.96-1.83). In MCBS, compared to participants with no trouble hearing, those with a lot of trouble hearing had 1.7 times the odds ( 95% CI = 1.150-2.623) of being dissatisfied. This has implications for patient-centered care planning given that Medicare ties reimbursement to patient-reported satisfaction.

